# Risk Factors and Predictive Parameters of Necrotizing Enterocolitis in Preterm Infants—A Single-Center Retrospective Study

**DOI:** 10.3390/diseases13110368

**Published:** 2025-11-10

**Authors:** Tamas Toth, Angela Borda, Reka Borka-Balas, Manuela Cucerea, Emoke Andrea Szasz, Horea Gozar, Radu-Alexandru Prisca

**Affiliations:** 1 Institution Organizing University Doctoral Studies (IOSUD), George Emil Palade University of Medicine Pharmacy Science and Technology of Targu Mures, 540142 Targu Mures, Romania; tamas.toth@umfst.ro; 2 Pediatric Surgery and Orthopedics Department, Targu Mures Emergency Clinical County Hospital, 540136 Targu Mures, Romania; 3 Histology Department, George Emil Palade University of Medicine Pharmacy Science and Technology of Targu Mures, 540142 Targu Mures, Romania; 4 Pediatric Clinic I, Targu Mures Emergency Clinical County Hospital, 540136 Targu Mures, Romania; 5 George Emil Palade University of Medicine Pharmacy, Science and Technology of Targu Mures, 540136 Targu Mures, Romania; 6 Neonatology Department, Targu Mures Emergency Clinical County Hospital, 540136 Targu Mures, Romania

**Keywords:** necrotizing enterocolitis, surgery, risk factors, prematurity, low birth weight, Bell stage

## Abstract

**Background and Objectives**: Necrotizing enterocolitis (NEC) represents a severe gastrointestinal emergency in preterm infants. The aim of this study was to identify risk factors and predictive parameters for NEC requiring surgery and to evaluate associated short-term outcomes. **Materials and Methods**: We conducted a retrospective study in preterm neonates diagnosed with NEC admitted to a tertiary neonatal intensive care unit (NICU) between January 2015 and May 2025. Demographic data, perinatal events, risk factors, clinical signs, imaging findings, and outcomes were analyzed, with a particular focus on surgically managed cases. Descriptive and inferential statistical methods were applied. **Results**: Forty-four infants met the inclusion criterion. The mean gestational age (GA) was 29.34 ± 4.3 weeks, and the mean birth weight was 1100 ± 563 g. According to Bell’s severity index, 45.5% had Bell Stage I, 36.4% Stage II, and 18.2% Stage III. Eleven patients (25%) required surgery. All surgical patients had abdominal distension, and 63.6% had bilious gastric residue. Abdominal X-ray showed pneumoperitoneum in 72.7% and pneumatosis intestinalis in 27.3% of cases. Laboratory abnormalities, including thrombocytopenia, elevated C-reactive protein (CRP) and lactate dehydrogenase (LDH), and hyponatremia (45.5%; 133 ± 6.95 mmol/L), were frequently associated with surgical NEC. A lower GA and birth weight correlated with a higher Bell stage (*p* = 0.0085 and *p* = 0.0291). Overall mortality was 29.5% (13/44); surgical mortality was 9.1% (1/11). **Conclusions**: In this single-center lot, low gestational age and birth weight, abdominal distension with bilious residuals, systemic inflammation, and hyponatremia were frequent among infants who required surgery. Selected infants may benefit from early surgery even without perforation, but inferences are limited by this study’s sample size and retrospective design. Prospective multi-center studies are needed to validate predictors and refine surgical timing.

## 1. Introduction

Necrotizing enterocolitis (NEC) represents a severe and life-threatening gastrointestinal emergency that primarily affects premature infants. Both short- and long-term sequelae are significant, including intestinal failure and neurodevelopmental impairment, reported in up to 25% of affected infants [1].

The prevalence of NEC varies between 1% and 11% in very low-birth-weight (VLBW < 1500 g) infants and may reach 22% in extremely low-birth-weight (ELBW < 1000 g) infants [2,3,4]. Despite advances in neonatal intensive care, mortality remains substantial. A systematic review by Jones et al. reported an overall mortality rate of 23.5% with a highest mortality rate of 50.9% among ELBW infants requiring surgical intervention [5].

A range of clinical and non-clinical risk factors have been associated with the development of NEC. These include preterm gestational age, low birth weight, congenital heart disease, bronchopulmonary dysplasia (BPD), retinopathy of prematurity (ROP), respiratory distress syndrome (RDS), neonatal resuscitation, mechanical ventilation, umbilical vein catheterization, transfusion of fresh frozen plasma, administration of surfactant, use of antibiotics, and vasoactive drugs [1].

The pathogenesis of NEC is multifactorial. Intestinal immaturity, inflammatory responses, dysbiosis, hypoxia–ischemia, and feeding practices contribute to mucosal injury and barrier failure, leading to bowel wall necrosis and potential perforation [6,7]. Formula feeding and altered microbial colonization have also been implicated, while breast milk is protective. Prenatal corticosteroid administration is associated with lower rates of NEC and can be considered a protective factor [6].

Diagnosis is based on clinical, laboratory, and radiologic findings.

Common clinical signs include abdominal distension, vomiting or gastric residuals, and hemorrhagic stools.

Laboratory abnormalities such as thrombocytopenia and elevated inflammatory markers are typical, and radiologic features may reveal pneumatosis intestinalis, portal venous gas, or fixed distended bowel loops.

Pneumoperitoneum is classically considered as an absolute surgical indication, although progressive clinical deterioration despite maximal medical therapy may also warrant surgical intervention [8].

Disease severity is classified by Bell’s staging system. Stage I (suspected NEC) shows non-specific symptoms (temperature instability, lethargy, apnea, and bradycardia), gastrointestinal manifestations (poor feeding, gastric residuum, mild abdominal distension, and occult blood in the stool), and mild ileus on the abdominal radiograph. Stage II (definite NEC) includes the mentioned symptoms above and occult or gross gastrointestinal bleeding, marked abdominal distension with ileus, small bowel separation, pneumatosis intestinalis, and portal vein gas imaging. Stage III NEC is an advanced phase with deterioration of vital signs, evidence of septic shock, and pneumoperitoneum on X-ray [9].

Although surgery is lifesaving in advanced NEC, it carries significant risks such as short bowel syndrome, growth restriction, and neurodevelopmental impairment. Therefore, identifying early predictors of surgical need before intestinal perforation or full-thickness necrosis occurs is essential to improve outcomes.

Single-center data provide valuable insights into local epidemiology, risk factors, and outcomes, which may differ across regions and health systems.

The aim of this study is to analyze risk factors for NEC in preterm infants and to evaluate clinical, laboratory, and radiological parameters that may indicate the need for early surgical intervention.

## 2. Materials and Methods

### 2.1. Study Sample and Design

We performed a single-center retrospective clinical study at the County Emergency Clinical Hospital Targu Mures, a tertiary-level NICU (neonatal intensive care unit) that serves as a regional referral center in Romania. Every year, it handles a high volume of critically ill neonates, including more than 250 premature newborns, of which around 60 infants are born at less than 32 weeks gestation, and receives both in-born and referred neonates from regional hospitals.

This study includes preterm infants diagnosed with NEC and admitted to the neonatal intensive care unit (NICU) between January 2015 and May 2025. This study was conducted in accordance with the Declaration of Helsinki and approved by the Institutional Review Board of County Emergency Clinical Hospital Targu Mures (approval no. 20406/23.07.2025). Cases were identified through electronic medical records.

The inclusion criterion was confirmed diagnosis of NEC during hospitalization.

Exclusion criteria included incomplete medical records or other diagnosed gastrointestinal disorders (meconium ileus, malrotation with volvulus, intestinal atresia, or spontaneous intestinal perforation).

### 2.2. Case Definition

NEC was diagnosed using combined clinical, laboratory, and radiographic findings, consistent with standard practice. Disease severity was classified according to Bell’s staging system, mentioning that across the 10-year period, examinations were not always performed by the same physicians and radiographic interpretations may have varied among radiologists.

### 2.3. Study Variables

Relevant data were collected from medical records and entered in a dedicated database. Variables included perinatal events, antenatal corticosteroid administration, mode of delivery, gestational age (GA), anthropometric measurements, infant medication, cardiologic comorbidities (atrial septal defect, persistent arterial duct, patent foramen ovale, and pulmonary hypertension), clinical features, treatment approaches, and final outcomes. Feeding data was not consistently recorded.

Fetal distress syndrome denoted signs of compromised oxygenation of the fetus in utero such as abnormal fetal heart rate tracing, reduced heart rate variability, or meconium-stained amniotic fluid, indicative of fetal hypoxia. Neonatal resuscitation was defined as any maneuvers to establish effective spontaneous breathing at birth. This could include stimulation, positive-pressure ventilation, endotracheal intubation, or chest compression as needed to achieve respiration. Mechanical ventilation referred to all forms of assisted ventilation, both invasive ventilation (endotracheal intubation with conventional mechanical ventilation) and non-invasive ventilatory support (nasal continuous positive airway pressure—CPAP or BiPAP).

### 2.4. Statistical Analysis

Data analysis included descriptive and inferential statistical methods. The Shapiro–Wilk test was used to assess the distribution of continuous variables. For comparisons between two groups, a Student’s *t*-test was applied for Gaussian distribution data, while the Mann–Whitney U test was used for non-Gaussian distributions. For comparisons involving more than two groups, ANOVA with a Bonferroni test was used for parametric data, and the Kruskal–Wallis test with Dunn’s multiple comparisons test was applied for non-parametric data.

Correlation analyses employed Pearson’s correlation coefficient for Gaussian-distributed variables, and Spearman’s rank correlation for non-Gaussian-distributed variables.

A *p*-value < 0.05 was considered statistically significant. All analyses were performed using SPSS software trial version 31.0 (IBM Corp., Chicago, IL, USA).

To explore independent predictors of neonatal mortality, we fitted a multivariable binary logistic regression including the clinically relevant variables used in this manuscript: gestational age, birth weight, fetal distress syndrome, neonatal resuscitation in the delivery room, cardiologic comorbidities, mechanical ventilation, fresh frozen plasma administration, and surgical intervention. Results are reported as odds ratios (ORs) with 95% confidence intervals and are displayed in a forest plot. Overall model performance was summarized with Nagelkerke R^2^. Given the limited number of deaths, estimates were interpreted cautiously and all *p*-values treated as exploratory without multiplicity adjustment.

## 3. Results

Forty-four infants met the inclusion criterion. The female-to-male ratio was 1:1.44. The mean gestational age was 29.34 ± 4.32 weeks, and the mean birth weight was 1100 ± 563 g. The youngest infant was born at 23 weeks, weighing 450 g, and was discharged after 120 days of hospitalization.

Among these, 20 infants (45.5%) were Bell Stage I, 16 (36.4%) were Bell Stage II, and 8 (18.2%) were Bell Stage III. A lower GA and birth weight correlated with a higher Bell stage (*p* = 0.0085; *p* = 0.0291), as is presented in Table 1. Infants in Bell Stage III had a higher mean birth weight than those in Stage II, which we interpret as a consequence of the small subgroup size rather than a true biological effect.

**Figure 1:**
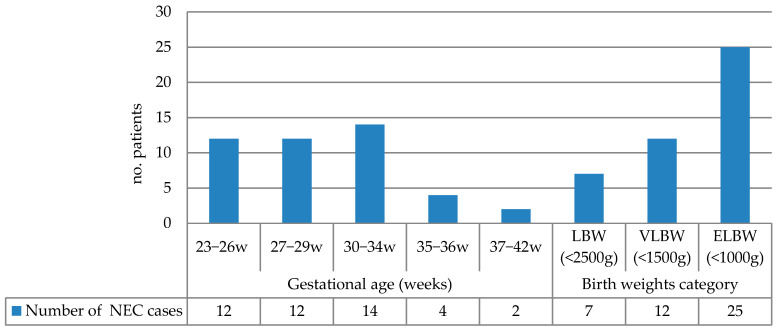
NEC incidence grouped by gestational age and birth weight (LBW: low birth weight; VLBW: very low birth weight; ELBW: extremely low birth weight).

The NEC incidence grouped by gestational age and birth weight is summarized in Figure 1. The highest incidence occurred in infants born at 30–34 weeks (31.81%), followed by the 23–26-week and 27–29-week groups (each 27.27%). Extremely low-birth-weight (ELBW) infants (<1000 g) accounted for 56.81% of NEC cases. Table 2 presents the correlation of various risk factors with birth weight and gestational age.

Significant correlations were observed between a lower median birth weight and prenatal corticosteroid administration (*p* = 0.0050), neonatal resuscitation (*p* = 0.0342), hemorrhagic stools (*p* = 0.0442), and cardiologic comorbidities (*p* = 0.0280). Similarly, mean gestational age was significantly lower in infants who received prenatal corticosteroids (*p* = 0.0002) and required neonatal resuscitation (*p* = 0.0100).

To further evaluate perinatal adaptation, the 1 min Apgar score was analyzed as a continuous variable. The mean value was 5.82 ± 2.29 and showed a moderate positive correlation with both gestational age (r = 0.56, *p* = 0.00007) and birth weight (r = 0.49, *p* = 0.00077). These correlations indicate that infants with a higher gestational age and birth weight tend to have higher Apgar scores and better immediate postnatal adaptation, consistent with their lower requirement for resuscitative maneuvers in the delivery room.

A lower median birth weight was significantly associated with increased exposure to antenatal corticosteroids, higher rates of neonatal resuscitation, occurrence of hemorrhagic stools, and cardiologic comorbidities. These interrelated factors represent both risk markers and prognostic indicators for adverse outcomes in NEC. The greater frequency of corticosteroid exposure and resuscitation maneuvers among lower-birth-weight infants reflects their degree of prematurity, underscoring their vulnerability to severe complications and poor prognosis.

A significant difference in median gestational age was observed between infants who died and those transferred to the premature infant care center (*p* = 0.0338, Dunn test), indicating that a lower gestational age is associated with higher mortality.

However, these correlations should be interpreted with caution due to the small sample size and retrospective design of this study, which limit its statistical power and do not allow adjustment for potential confounders. Table 3 presents the associations between birth weight, gestational age, and clinical signs.

The median postnatal day of NEC onset in our group was after 15.5 (range 6–38) days. Earlier NEC onset was significantly associated with lower gestational age and birth weight (*p* < 0.001 and *p* < 0.001, respectively). NEC occurred significantly earlier in infants who required resuscitation at birth (median 9 vs. 29 days, *p* = 0.008). Although infants with fetal distress syndrome also showed an earlier onset tendency (median 8.5 vs. 16.5 days), this was not statistically significant (*p* = 1.00). These findings (Table 4) suggest that earlier NEC onset is primarily driven by prematurity and perinatal instability, reflected by the need for resuscitation.

We also explored whether the timing of NEC onset correlated with survival outcomes. Among the non-surgical infants, those who died developed NEC significantly earlier (median 9 days) compared with survivors (median 25.5 days), although this difference did not reach statistical significance (*p* = 0.26). In the surgical group, onset timing was similar between the single fatal case (9 days) and survivors (median 8 days). These findings suggest that early-onset NEC may be associated with increased vulnerability and mortality, particularly among non-surgical patients.

**Figure 2:**
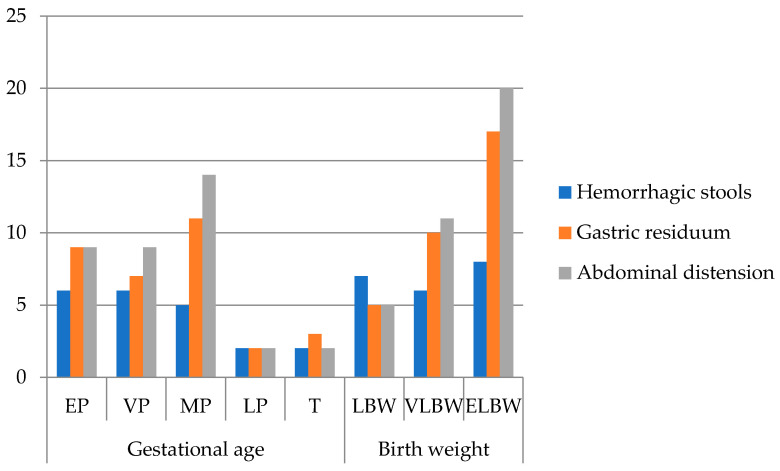
Frequency of clinical signs of all patients (n = 44) grouped by gestational age and birth weight (EP: extremely premature—23–26 w; VP: very premature—27–29 w; MP: moderately premature—30–34 w; LP: late premature—35–36 w; T: term—37–42 w; LBW: low birth weight < 2500 g; VLBW: very low birth weight < 1500 g; ELBW: extremely low birth weight < 1000 g; no. patients = number of patients).

The most common clinical signs in our patients (Figure 2) were abdominal distension (81.8%), gastric residuals (72.7%), and hemorrhagic stools (47.7%), consistent with classical NEC presentations. One patient presented with acute scrotum, later confirmed intraoperatively as NEC. Extremely premature infants showed fewer documented clinical signs, likely due to early mortality and rapid deterioration limiting observation rather than true differences in presentation.

**Figure 3:**
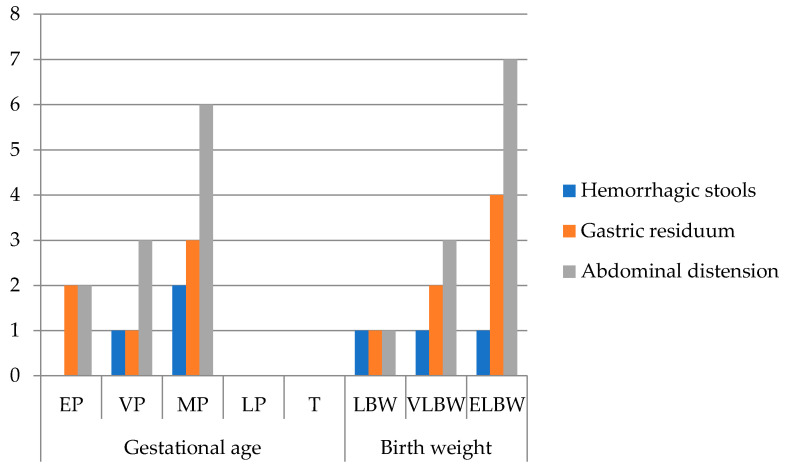
Frequency of clinical signs in surgical NEC (n = 11) grouped by gestational age and birth weight (EP: extremely premature—23–26 w; VP: very premature—27–29 w; MP: moderately premature—30–34 w; LP: late premature—35–36 w; T: term—37–42 w; LBW: low birth weight < 2500 g; VLBW: very low birth weight < 1500 g; ELBW: extremely low birth weight < 1000 g; no. patients = number of patients).

Out of 44 patients, 25% (n = 11) required surgical intervention based on their Bell stages (Figure 3).

The mean birth weight and gestational age in the surgical group were 1086 g and 29.45 ± 2.81 weeks, respectively. Most neonates in this group were vaginally delivered (63.6%), had a monitored pregnancy (72.7%), and presented respiratory distress syndrome at birth (81.8%). Neonatal resuscitation was required in 90.9%, and 45.5% received mechanical ventilation during hospitalization, though none developed bronchopulmonary dysplasia. Retinopathy of prematurity was observed in 54.5%, and 36.4% had cardiologic comorbidities. Central vascular access via umbilical vein catheterization was performed in 90.9% of cases. Administration of fresh frozen plasma and pentoxifylline occurred in 63.6%, and vitamin K derivative in 72.7%.

All surgically managed infants presented abdominal distension, 63.6% had bilious gastric residuum, and 27.3% had hemorrhagic stools. Laboratory findings included thrombocytopenia, markedly elevated lactate dehydrogenase (LDH), and increased C-reactive protein (CRP). Hyponatremia was noted in 45.5%, with a mean plasma sodium level of 133.18 ± 6.95 mmol/L. Abdominal X-ray revealed pneumoperitoneum in 72.7% and pneumatosis intestinalis in 27.3%. Median time from NEC diagnosis to surgery was 5 days.

Outcomes (death, transfer to the premature infant care center, or discharge home) were recorded for all 44 infants included in this study.

The outcome in the surgical group was 9.1% mortality (n = 1) and 27.27% discharge home with multidisciplinary follow-up. Infants were transferred to the premature infant care center for continued care in 63.6% of the cases when they were considered healed from the NEC episode without a need for intensive therapy, but they had to reach the weight of 2500 g as a criterion for discharge.

The outcome in the overall study sample was mortality rate of 29.5% (n = 13), transfer to premature infant care center in 45.45% of patients (n = 20), and 25% (n = 11) discharged home in healthy condition. In the group of deceased newborns (n = 13) the mean gestational age was 28.92 weeks and mean birth weight was 1061 g, 23.1% had respiratory distress syndrome at birth and 53.8% required neonatal resuscitation, 69.2% had abdominal distension, 76.9% bilious gastric residuum, and 38.5% had hemorrhagic stools. Mechanical ventilation was required in 38.46%, with no cases of bronchopulmonary dysplasia. Retinopathy of prematurity and cardiologic comorbidities were documented in 30.8% and 15.4%, respectively. Umbilical vein catheterization was performed in 61.5%. Administration of fresh frozen plasma and pentoxifylline was recorded in 23.1% and 38.5% of cases, respectively.

**Figure 4:**
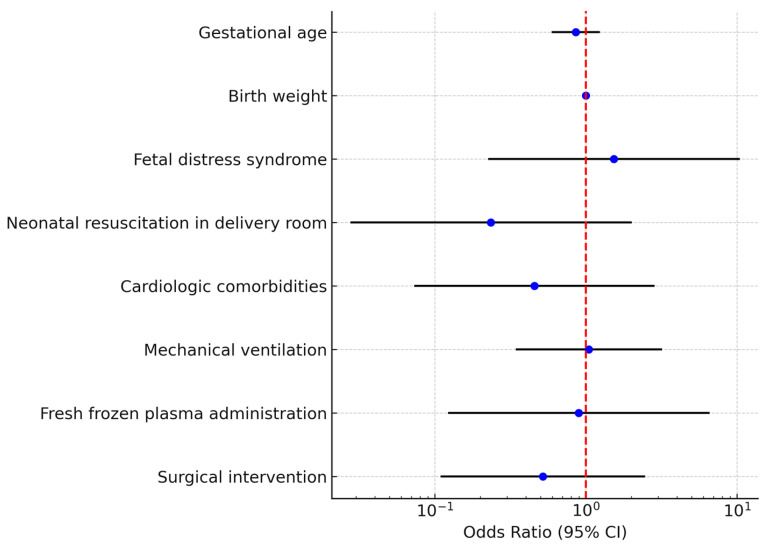
Multivariate logistic regression analysis for mortality in neonates with necrotizing enterocolitis (NEC)The forest plot displays the odds ratios (ORs) and corresponding 95% confidence intervals (CIs) for each variable included. Each blue dot represents the point estimate of the OR for the corresponding predictor, while the horizontal black lines indicate the 95% CIs. The red dashed vertical line marks the reference value of OR = 1, which represents no effect on mortality. Variables with confidence intervals crossing the red line are not statistically significant, whereas those entirely to one side of the line indicate a significant association.

A binary logistic regression model was constructed including gestational age, birth weight, fetal distress syndrome, resuscitation in the delivery room, cardiologic comorbidities, mechanical ventilation, fresh frozen plasma administration, and surgical intervention. The overall model was not statistically significant (χ^2^ test *p* > 0.05) and explained approximately 23% of the variance (Nagelkerke R^2^ = 0.23). None of the predictors reached statistical significance as independent risk factors for death (Table 5, Figure 4).

## 4. Discussion

NEC typically manifests during the second and third weeks of life, with an inverse relationship observed between gestational age, birth weight, and the risk of disease onset [10]. Sharma et al., in a cohort of 202 infants, reported NEC incidence rates of 24% in extremely premature (23–26 weeks), 33% in very premature (27–29 weeks), 28% in moderately premature (30–34 weeks), 28% in late preterm (35–36 weeks), and 9% in term infants (37–42 weeks) [11]. Our findings are consistent with these data: 27.3% of our patients were extremely premature (GA < 26 weeks) and 56.8% had an extremely low birth weight. Prematurity, along with its associated low gestational age and birth weight, remains the principal predisposing factor for NEC.

### 4.1. Comorbidities and Systematic Inflammatory Processes

Samuels et al. highlighted the difficulty in establishing clear causality in NEC due to complex multifactorial pathophysiology [1].

Inflammatory processes are central mechanisms not only in NEC but also in other prematurity-related complications such as bronchopulmonary dysplasia (BPD), retinopathy of the prematurity (ROP), and cystic periventricular leukomalacia [12]. In our cohort, 47.7% of NEC cases were associated with ROP, and 18.2% developed BPD. While the literature reports that 25% of infants with surgical NEC develop BPD, none of our surgical NEC cases presented this complication [13].

### 4.2. Respiratory Morbidity and Interventions

Low GA was also associated with increased incidence of respiratory distress syndrome, transfusion requirements, and ventilator dependence [14].

In our study, 23 (52.3%) out of 44 infants developed respiratory distress syndrome, of whom 20 (45.5%) required mechanical ventilation. Although surfactant therapy is not a direct risk factor for NEC, its necessity in severe respiratory distress syndrome may reflect disease severity and contribute indirectly. Mechanical ventilation itself has been associated with increased NEC risk [15].

### 4.3. Cardiac Comorbidities

Cardiac comorbidities—such as patent ductus arteriosus (PDA), atrial septal defect, and pulmonary hypertension—are more frequent in growth-restricted and preterm infants and are recognized contributors to NEC development [14,16].

We found a statistically significant association between birth weight and cardiologic comorbidities (*p* = 0.0280), with PDA present in five cases. None of our surgical NEC patients had cyanotic heart disease, although the literature suggests a 24.8% prevalence in the surgical NEC population, likely due to altered hemodynamics and nutrient absorption [14].

### 4.4. Perinatal Interventions and Therapeutic Exposures

We identified a significant correlation between the gestational age, birth weight, and prenatal corticosteroid administration (*p* = 0.0002 and *p* = 0.005, respectively) and the need for neonatal resuscitation maneuvers (*p* = 0.01, *p* = 0.034). This association reflects a greater prematurity and clinical indication for corticosteroids, rather than indicating that corticosteroids are a direct risk factor for NEC. Preterm infants are more likely to require resuscitative interventions.

Consistent with these findings, the 1 min Apgar score showed a moderate positive correlation with both gestational age and birth weight, supporting that more mature infants have better immediate postnatal adaptation. This relationship further underscores that perinatal compromise and physiological immaturity simultaneously contribute to NEC vulnerability.

Antenatal corticosteroids have been shown to reduce the incidence of NEC, RDS, and intraventricular hemorrhage [17,18]. In our sample, 50% (n = 22) of cases received antenatal corticosteroids. Among them, 63.6% developed RDS, 13.6% (n = 3) had intraventricular hemorrhage, and the mortality rate within this subgroup was 18.2% (n = 4), lower than the overall rate. The required resuscitation rate was 27.3% (n = 6).

Cesarean section has been proposed as a protective factor against NEC due to decreased perinatal stress [15]. In our cohort, 47.7% (n = 21) of cases were delivered via cesarean section, and 61.90% (n = 13) of those had fetal distress syndrome.

Prolonged antibiotic exposure has been implicated in NEC pathogenesis. Alexander et al. reported a threefold increase in NEC risk following more than 10 days of broad-spectrum antibiotic use [19]. In our study, 97.7% of infants received antibiotics for longer than 10 days. Most commonly, initial therapy consisted of Ampicillin–sulbactam and Amikacin (n = 35). Other regimens included Meropenem, Colistin, Vancomycin, and Metronidazole, particularly in cases with suspected anaerobic infections or lack of response.

Beyond gestational age and corticosteroid exposure, perinatal oxygenation events also appeared to influence NEC risk.

In our group, fetal distress syndrome occurred in smaller and early premature infants, whereas the need for resuscitation at birth was more common among neonates with a higher gestational age and birth weight. This apparent paradox likely reflects distinct underlying mechanisms. Fetal distress syndrome represents a chronic antenatal hypoxic environment leading to intrauterine growth restriction and a lower birth weight.

The requirement for resuscitation in the delivery room reflects an acute perinatal hypoxic–ischemic event that may affect otherwise more mature infants. The physiological redistribution of blood flow to vital organs during such acute asphyxia could transiently compromise intestinal perfusion, potentially contributing to NEC pathogenesis [20,21].

The impact of perinatal anoxia may also explain the relatively weak association between gestational age < 31 weeks and NEC incidence. While immaturity remains a major predisposing factor, acute perinatal hypoxic episodes may trigger NEC independently of extreme prematurity. Supporting this interpretation, our analysis showed that infants who required resuscitation at birth developed NEC significantly earlier than those who did not (median 9 vs. 29 days, *p* = 0.008), whereas the presence of fetal distress syndrome alone did not reach statistical significance. These results suggest that perinatal asphyxia accelerates disease onset, highlighting the complex interaction between gestational immaturity and postnatal ischemic–inflammatory injury [22].

### 4.5. Diagnosis and Management of NEC

Diagnosis was based on clinical, laboratory, and radiologic findings. Case severity stratification was made using Bell’s staging system. The inverse correlation between birth weight, gestational age, and NEC severity is well documented [23]. We also found statistically significant correlations between the Bell stage and birth weight and gestational age (Kruskal–Wallis test: *p* = 0.0291 and *p* = 0.0085, respectively).

Classically defined by Mizrahi et al., NEC presents with vomiting, abdominal distension, shock, and gastrointestinal bleeding [24].

Abdominal distension and gastric residuals were the most frequent early signs, whereas hemorrhagic stools occurred in 48% of patients. Interestingly, in extremely premature infants, the frequency of these clinical signs appeared lower compared to moderately premature infants. This finding may reflect rapid clinical deterioration and early mortality, limiting the window for symptom documentation—a phenomenon also reported in other studies [14].

NEC’s initial signs may be non-specific, including feeding intolerance, lethargy, and temperature instability.

One of our cases presented with acute scrotum syndrome due to herniation of inflammatory peritoneal fluid into the scrotum, mimicking testicular pathology. Awareness of this atypical presentation is essential for differential diagnosis in male infants.

We also identified a statistically significant association between low birth weight and the presence of hemorrhagic stools (*p* = 0.044), an early sign of NEC or other gastrointestinal disorders. This is consistent with reports indicating a higher incidence of GI bleeding in preterm low-birth-weight infants [25].

Radiologic diagnosis of NEC is typically made via abdominal X-ray, revealing pneumatosis intestinalis, portal venous gas, or pneumoperitoneum, though the latter is not always present in bowel perforation or necrosis [26].

In our 11 surgical NEC cases, 8 had pneumoperitoneum, and 3 exhibited pneumatosis intestinalis, and consequently, 72.7% had Bell Stage III and 27.3% had Bell Stage II. Given the relative surgical indications, we proceeded with surgery in the three Bell II cases, as they showed clinical deterioration despite receiving maximal medical management. The outcome of these patients was favorable, but these results should be interpreted cautiously given the small sample size.

Laboratory markers such as CRP, LDH, leukocytosis, and thrombocytopenia are valuable indicators of disease severity. All surgical patients had elevated CRP and LDH levels, as well as thrombocytopenia.

Hyponatremia is common among preterm infants and may reflect underlying disease progression. In our surgical group, 45.5% of infants had hyponatremia with a mean sodium level of 133 ± 6.95 mmol/L. Prior studies suggest that hyponatremia <130 mmol/L may indicate advanced disease, warranting close monitoring [27,28].

The decision for surgery remains challenging. Clinical indicators include apnea, bradycardia, temperature instability, abdominal distension and tenderness, erythema, hemorrhagic stools, and systemic deterioration [22,29]. In our cohort, the mean time from symptom onset to surgery was five days—possibly due to a reliance on pneumoperitoneum as the definitive surgical trigger.

Garcia et al. identified perinatal RDS, hyperglycemia, coagulopathy, and neutrophilia as predictors of surgical NEC [30]. Our surgical patients frequently presented with respiratory distress (81.8%), neonatal resuscitation (90.9%), umbilical vein catheterization (90.9%), abdominal distension (100%), and gastric residuals (63.6%).

### 4.6. Outcome and Follow-Up

Surgical intervention in NEC patients heightens the risk of complications, including short bowel syndrome, neurodevelopmental impairment, and growth failure [31]. We do not have data on long-term follow-up to know the occurrence rate of these complications. In our center, postoperative follow-up is typically performed by pediatric gastroenterologists, and patients are referred back to our surgical team only if complications arise. Consequently, our data may not fully capture the long-term outcomes of these infants.

Earlier NEC onset was significantly associated with a lower gestational age and birth weight, consistent with prior studies demonstrating that disease tends to occur sooner in smaller and early immature infants. Kaplina et al. emphasize that hypoxia and hypoperfusion are recognized contributors for NEC, but prematurity and low birth weight remain the primary risk factors for NEC onset and severity [32].

In our cohort, overall mortality was 29.5%, whereas only 9.1% of surgically treated infants died. This differed from many published series, where surgical NEC often carries the highest mortality. This may be explained by early deaths among non-operated infants, reflecting both illness severity at presentation and the small sample size. Therefore, the apparent survival advantage in the surgical subgroup should be interpreted cautiously.

In our multivariate model, no independent predictor of neonatal mortality reached statistical significance, consistent with the limited sample size and wide confidence intervals. Nonetheless, a higher gestational age and surgical intervention appeared to reduce mortality risk, while intrauterine distress and invasive ventilation showed a trend toward increased mortality. These findings are consistent with prior studies suggesting that mortality in NEC is influenced by the degree of prematurity, comorbidities, and the infant’s clinical trajectory rather than a single isolated parameter. The lack of significance in our model highlights the need for larger, multi-center datasets to clarify independent risk factors.

To determine whether timing of NEC onset influenced survival, we compared the postnatal day of NEC onset between survivors and non-survivors. Among non-surgical infants, those who died developed NEC markedly earlier (median 9 days) than survivors (median 25.5 days), although this difference did not reach statistical significance (*p* = 0.26). In the surgical group, the single death occurred at a similar onset time (9 days) compared with survivors (median 8 days). These findings support the hypothesis that earlier-onset NEC reflects greater physiological instability, which may contribute to the higher mortality observed in non-surgical infants, whereas later-onset cases often occur in more stabilized neonates who can tolerate surgical intervention [33,34].

### 4.7. Strengths and Limitations

This study provides valuable insight into the presentation, management, and outcomes of NEC in a tertiary NICU. The decade-long study period allowed us to examine temporal trends in management and outcomes, while also providing a comprehensive overview of the clinical, laboratory, and radiological parameters associated with NEC severity and surgical intervention.

However, several limitations of our study should be acknowledged. Its retrospective, single-center design and relatively small sample size inherently limit the statistical power of the analysis and restrict the generalizability of the findings.

The Apgar score was evaluated as an indicator of perinatal adaptation and correlated strongly with gestational age and birth weight, reflecting overlapping predictive information already captured by these parameters.

In addition, changes in clinical management over the ten-year period and occasional inconsistencies in medical records made it impossible to obtain a more exhaustive dataset, particularly for important variables such as feeding type and other modifiable risk factors. Finally, long-term follow-up data, including gastrointestinal complications and neurodevelopmental outcomes, were not consistently available.

Despite these limitations, our findings contribute to the understanding of NEC in preterm infants, particularly regarding clinical, laboratory, and imaging features that may inform surgical decision-making. Nevertheless, given the small cohort and retrospective design, these associations should be interpreted as exploratory rather than definitive predictors. A future prospective study will aim to clarify the issues raised by the presented data, as well as all of the shortcomings of retrospective data.

## 5. Conclusions

Necrotizing enterocolitis remains a life-threatening condition in preterm infants, particularly among those with a lower gestational age and birth weight. Our results suggest that surgical intervention may be beneficial in selected infants, even without radiologic evidence of perforation, when guided by a combination of clinical, laboratory, and imaging findings. Our findings also indicate that NEC tends to occur earlier in infants with a lower gestational age, a lower birth weight, and evidence of perinatal compromise such as fetal distress or need for resuscitation. This earlier onset likely reflects greater physiological instability and may contribute to the higher mortality observed among non-surgical infants.

The small sample size, retrospective design, and single-center setting of this study substantially limit the generalizability of these findings. The observed survival advantage in surgically managed infants likely reflects selection bias and should not be over-interpreted. Future prospective, multi-center studies with standardized protocols and long-term follow-up are required to refine risk stratification, identify predictor factors for surgical treatment, and optimize outcomes in this vulnerable population. 

## Figures and Tables

**Table 1:** Correlation between Bell’s staging system and birth weight and gestational age.

		Birth Weight in Grams			Gestational Age in Weeks		
		Mean± SD	Median	*p*-Value	Mean ± SD	Median	*p*-Value
Bell’s Staging System	Stage I (*n = 20*)	1345 ± 702.6	1200	**0.0291**	31.00 ± 4.554	30.50	**0.0085**
	Stage II (*n = 16*)	850.0 ± 268.2	825.0		26.88 ± 3.160	26.50	
	Stage III (*n = 8*)	1085 ± 335.6	965.0		30.13 ± 2.850	30.00	

Statistically significant difference *p* < 0.05.

**Table 2:** Correlation between birth weight, gestational age, and risk factors for NEC.

		Birth Weight in Grams			Gestational Age in Weeks		
		Mean ± SD	Median	*p*-Value	Mean ± SD	Median	*p*-Value
Gender	Female (n = 18)	1241 ± 758.2	990.0	0.7378	29.83 ± 4.706	28.50	0.5238
	Male (n = 26)	1032 ± 357.6	965.0		29.00 ± 3.868	28.00	
Delivery mode	Vaginal (n = 23)	1137 ± 466.4	1000	0.3968	29.65 ± 4.238	28.00	0.6126
	Cesarean n = 21)	1097 ± 657.0	980.0		29.00 ± 4.231	29.00	
Prenatal corticosteroid administration	Yes (n = 22)	876.4 ± 272.6	800.0	**0.0050**	27.14 ± 2.833	27.00	**0.0002**
	No (n = 22)	1359 ± 665.9	1240		31.55 ± 4.228	32.50	
Fetal distress syndrome	Yes (n = 10)	840.0 ± 275.8	850.0	0.0512	28.90 ± 3.814	30.00	0.7103
	No (n = 34)	1199 ± 596.8	1025		29.47 ± 4.350	28.00	
Respiratory distress syndrome	Yes (n = 23)	960.0 ± 249.4	950.0	0.2166	28.57 ± 3.145	28.00	0.2032
	No (n = 21)	1290 ± 737.1	1200		30.19 ± 5.056	29.00	
Neonatal resuscitation in delivery room	Yes (n = 34)	1206 ± 593.1	1025	**0.0342**	30.21 ± 4.220	30.00	**0.0100**
	No (n = 10)	819.0 ± 273.1	825.0		26.40 ± 2.547	26.50	
Surfactant administration	Yes (n = 16)	947.5 ± 262.6	935.0	0.3164	27.88 ± 3.008	27.50	0.0798
	No (n = 28)	1215 ± 657.5	1030		30.18 ± 4.587	30.00	
Mechanical ventilation	Yes (n = 20)	1102 ± 483.8	1000	0.9529	28.95 ± 3.900	28.00	0.5789
	No (n = 24)	1131 ± 624.8	965.0		29.67 ± 4.488	29.50	
Bronchopulmonary dysplasia	Yes (n = 8)	1151 ± 317.6	1140	0.3606	30.88 ± 3.563	30.50	0.1650
	No (n = 36)	1110 ± 602.8	950.0		29.00 ± 4.296	28.00	
Retinopathy of Prematurity	Yes (n = 21)	1091 ± 619.4	980.0	0.5251	28.52 ± 3.669	28.00	0.1673
	No (n = 23)	1142 ± 510.5	1080		30.09 ± 4.582	30.00	
Cardiologic comorbidities	Yes (n = 17)	1381 ± 723.1	1200	**0.0280**	30.53 ± 4.543	30.00	0.1380
	No (n = 27)	951.9 ± 347.9	890.0		28.59 ± 3.866	28.00	
Intraventricular hemorrhage	Yes (n = 6)	855.0 ± 280.3	875.0	0.2305	28.30 ± 4.030	27.50	0.5347
	No (n = 37)	1159 ± 582.7	980.0		29.50 ± 4.250	28.50	
Umbilical vein catheterization	Yes (n = 31)	1029 ± 348.0	1000	0.7967	28.94 ± 3.932	28.00	0.3284
	No (n = 13)	1329 ± 864.9	950.0		30.31 ± 4.803	28.00	
Vasoactive substance administration	Yes (n = 13)	1076 ± 547.4	950.0	0.6428	28.92 ± 4.499	28.00	0.6742
	No (n = 31)	1135 ± 571.8	980.0		29.52 ± 4.130	28.00	
Fresh frozen plasma administration	Yes (n = 15)	1017 ± 357.7	980.0	0.7566	28.67 ± 4.18	28.00	0.4501
	No (n = 29)	1170 ± 638.5	980.0		29.69 ± 4.235	28.00	
Phytonadione administration	Yes (n = 27)	1061 ± 442.1	980.0	0.6993	29.04 ± 3.868	28.00	0.5514
	No (n = 17)	1208 ± 712.8	950.0		29.82 ± 4.760	28.00	
Pentoxifylline administration	Yes (n = 26)	1054 ± 401.9	980.0	0.6932	29.65 ± 3.867	30.00	0.3878
	No (n = 18)	1210 ± 733.1	975.0		28.89 ± 4.714	27.50	
Surgical intervention	Yes (n = 11)	1086 ± 289.3	1000	0.5595	29.45 ± 2.806	30.00	0.9189
	No (n = 33)	1128 ± 627.1	950.0		29.30 ± 4.606	28.00	
Outcome	Death (n = 13)	1062 ± 890.9	750.0	0.0529	28.31 ± 5.360	28.00	0.4048
	Premature infant care center (n = 20)	1098 ± 301.2	1040		29.45 ± 3.471	28.50	
	Home (n = 11)	1221 ± 449.4	1050		30.36 ± 3.982	30.36	

Statistically significant difference *p* < 0.05.

**Table 3:** Correlations between birth weight, gestational age, and clinical signs.

		Birth Weight in Grams			Gestational Age in Weeks		
		Mean ± SD	Median	*p*-Value	Mean ± SD	Median	*p*-Value
Hemorrhagic stools	Yes (n = 21)	1299 ± 650.7	1280	**0.0442**	30.62 ± 4.674	30.00	0.0525
	No (n = 23)	952.2 ± 407.1	950.0		28.17 ± 3.407	28.00	
Gastric residuum	Yes (n = 32)	1153 ± 567.7	990.0	0.3910	29.63 ± 4.210	29.50	0.4701
	No (n = 12)	1023 ± 547.3	950.0		28.58 ± 4.252	27.00	
Abdominal distension	Yes (n = 36)	1080 ± 381.5	990.0	0.5122	29.56 ± 3.79	29.50	0.2390
	No (n = 8)	1288 ± 1073	875.0		28.38 ± 5.927	27.00	

Statistically significant difference *p* < 0.05.

**Table 4:** Correlation of NEC onset timing with perinatal factors.

	Correlation (ρ)/Median	*p*-Value
Gestational age (weeks)	−0.86	<0.001
Birth weight (g)	−0.74	<0.001
Fetal distress syndrome	16.5/8.5 days	1.00
Neonatal resuscitation in delivery room	29.0/9.0 days	0.008

**Table 5:** Multivariate logistic regression for predictors of neonatal mortality.

	OR (Exp(B))	95% C.I. for EXP(B)		*p*-Value
		Lower	Upper	
Gestational age	**0.855**	0.601	1.217	0.385
Birth weight	1.001	0.999	1.004	0.254
Fetal distress syndrome	1.534	0.229	10.270	0.659
Neonatal resuscitation in delivery room	0.235	0.028	1.978	0.183
Cardiologic comorbidities	0.457	0.074	2.809	0.398
Mechanical ventilation	1.046	0.348	3.142	0.937
Fresh frozen plasma administration	0.897	0.124	6.494	0.914
Surgical intervention	0.519	0.111	2.427	0.405

Odds ratios (ORs) with 95% confidence intervals (CIs) are shown.

## Data Availability

Data is contained within the article. Further inquiries can be directed to the corresponding author.

## Abbreviations

The following abbreviations are used in this manuscript:

BPD	Bronchopulmonary dysplasia
CRP	C-reactive protein
ELBW	Extremely low birth weight
EP	Extremely premature
GA	Gestational age
LDH	Lactate dehydrogenase
LP	Late premature
MP	Moderately premature
NEC	Necrotizing enterocolitis
NICU	Neonatal intensive care unit
PDA	Patent ductus arteriosus
ROP	Retinopathy of prematurity
RDS	Respiratory distress syndrome
T	Term
VLBW	Very low birth weight
VP	Very premature
